# Strategies for Hypothermia Compensation in Altricial and Precocial Newborn Mammals and Their Monitoring by Infrared Thermography

**DOI:** 10.3390/vetsci9050246

**Published:** 2022-05-23

**Authors:** Karina Lezama-García, Daniel Mota-Rojas, Julio Martínez-Burnes, Dina Villanueva-García, Adriana Domínguez-Oliva, Jocelyn Gómez-Prado, Patricia Mora-Medina, Alejandro Casas-Alvarado, Adriana Olmos-Hernández, Paola Soto, Ramon Muns

**Affiliations:** 1 PhD Program in Biological and Health Sciences [Doctorado en Ciencias Biológicas y de la Salud], Universidad Autónoma Metropolitana (UAM), Mexico City 04960, Mexico; 2192801631@alumnos.xoc.uam.mx; 2 Neurophysiology, Behavior and Animal Welfare Assessment, DPAA, Universidad Autónoma Metropolitana (UAM), Mexico City 04960, Mexico; 2212801915@alumnos.xoc.uam.mx (A.D.-O.); 2173025424@alumnos.xoc.uam.mx (J.G.-P.); 2211801831@alumnos.xoc.uam.mx (A.C.-A.); 2183072088@alumnos.xoc.uam.mx (P.S.); 3 Animal Health Group, Facultad de Medicina Veterinaria y Zootecnia, Universidad Autónoma de Tamaulipas, Victoria City 87000, Tamaulipas, Mexico; jmburnes@docentes.uat.edu.mx; 4 Division of Neonatology, National Institute of Health, Hospital Infantil de México Federico Gómez, Doctor Márquez 162, Mexico City 06720, Mexico; dinavg21@yahoo.com; 5 Department of Livestock Science, FESC, Universidad Nacional Autónoma de México (UNAM), Cuautitlán Izcalli 54714, Mexico; morapat@cuautitlan.unam.mx; 6 Division of Biotechnology—Bioterio and Experimental Surgery, Instituto Nacional de Rehabilitación-Luis Guillermo Ibarra Ibarra (INR-LGII), Mexico City 14389, Mexico; solmos@inr.gob.mx; 7 Agri-Food and Biosciences Institute, Livestock Production Sciences Unit, Hillsborough BT26 6DR, Northern Ireland, UK; ramon.muns@afbini.gov.uk

**Keywords:** thermoregulation, body temperature, brown adipose tissue, neonate welfare, shivering, vasoconstriction

## Abstract

Thermoregulation in newborn mammals is an essential species-specific mechanism of the nervous system that contributes to their survival during the first hours and days of their life. When exposed to cold weather, which is a risk factor associated with mortality in neonates, pathways such as the hypothalamic–pituitary–adrenal axis (HPA) are activated to achieve temperature control, increasing the circulating levels of catecholamine and cortisol. Consequently, alterations in blood circulation and mechanisms to produce or to retain heat (e.g., vasoconstriction, piloerection, shivering, brown adipocyte tissue activation, and huddling) begin to prevent hypothermia. This study aimed to discuss the mechanisms of thermoregulation in newborn domestic mammals, highlighting the differences between altricial and precocial species. The processes that employ brown adipocyte tissue, shivering, thermoregulatory behaviors, and dermal vasomotor control will be analyzed to understand the physiology and the importance of implementing techniques to promote thermoregulation and survival in the critical post-birth period of mammals. Also, infrared thermography as a helpful method to perform thermal measurements without animal interactions does not affect these parameters.

## 1. Introduction

Neonatal mortality in domestic animals such as lambs, calves, foals, piglets, and rodents responds to several maternal and offspring factors. Within these, hypothermia caused by excessive heat loss or inhibition of thermoregulation and heat production is considered a major element that causes mortality of newborn animals [1,2,3]. Hypothermia is mainly the result of starvation when the offspring is unable to suckle [1]. Neonatal survival within the first 24 to 72 hours is highly related to decreased body temperature experienced at birth. At this moment, the offspring abruptly transitions from a warm, nutritious, sterile, and controlled environment in utero to the extrauterine environment, usually at a much colder temperature (1 to 2 °C below), exposed to novel microorganisms and deprived of a nutrient supply via placenta [4,5,6,7]. These physical and physiological changes can generate hypoglycemia, hypoalbuminemia, energy alterations, or acid-base alterations leading to growth retardation or multiorgan failure [8].

The separate evolution of endothermy in mammals and birds is considered an important transition in vertebrate evolution. It is a unique case of convergence between these two groups, essential to their rapid spread across the planet and their ecological success [9,10]. Rezende et al. [10] observed that when metabolism increases, the sizes of the individuals decrease (and that is why dinosaurs gave rise to birds). In endotherm animals, thermoregulation is a homeostatic and dynamic process between the internal response of an organism and its external environment [11,12,13]. This internal response triggers a series of thermoregulatory mechanisms that are modulated by the preoptic area of the hypothalamus (POA) [14], and they are aimed at promoting energy conservation in the neonate, using this mechanism for growth, development, and cellular functions [15,16]. However, newborn’s interspecies characteristics, such as the presence or absence of thermogenic cells, e.g., brown adipocyte tissue its acronym is BAT; the presence of fur at birth, the thickness of the dermis, behavior at birth, locomotor abilities, and general organ development, can either facilitate or hinder thermoregulation [17].

These interspecies differences respond to physiological maturity, known as the newborn’s capacity to cope with the transition between the intrauterine life and the external environment. Such maturity involves activating several neuroendocrinological and behavioral changes. Nonetheless, nutrition, genetic selection, and pharmacology can also influence maturity at birth. Similarly, factors leading to the activation of the hypothalamic–pituitary–adrenal axis (HPA) [18], increasing the catecholamine and cortisol concentrations either in the fetus or the newborn animal [19], will cause changes in blood flow that will ultimately compromise the newborn’s thermoregulation capacity [20]. 

Determined mainly by physiological maturity, mammals can be classified into altricial and precocial species (see Section 3). Although altricial and precocial newborns have several mechanisms to maintain a stable body temperature [21,22], a sudden drop in temperature experienced at birth reduces vigor and affects their feeding ability. Consequently, the acquisition of immunoglobulins and the ingestion of nutrients that fuel thermogenesis are compromised [23,24,25,26,27,28].

Thermoregulation is one of the most complex mechanisms of the organism and a critical factor for the survival of newborn non-human mammals. However, it is not always well understood due to interspecies differences. Therefore, the present review aims to discuss the mechanisms of thermoregulation in domestic mammalian newborns, highlighting the differences between altricial and precocial species. The main thermoregulation mechanisms among domestic mammals (i.e., use of BAT activation, shivering, dermal vasomotor control, and thermoregulatory behaviors) will be analyzed to understand their physiology. Additionally, the importance of implementing diverse techniques, such as infrared thermography, that evaluate and promote thermoregulation and survival in the critical post-birth period of mammals will be reviewed.

## 2. General Thermoregulatory Mechanisms Triggered at Birth

In many species, the drop in temperature experienced at birth induces an immediate compensatory response by the neonate to modify the physiological parameters and to avoid further heat loss. It has been described that when the neonate experiences a drop of up to 2 °C in body temperature—in the case of piglets and ruminants—the body is triggered to use thermogenesis mechanisms coordinated by the Nervous System (NS) [25,29,30]. The thermoneutral zone is the temperature range in which an animal’s body temperature remains within the normal physiological range, being able to regulate either heat loss or heat production with minimal effort [31]. Further, endothermy or homeothermy is a state associated with thermogenesis, initiated by hypothalamic activation, which aims to reduce heat loss or increase heat production [32,33].

Exposure to cold temperatures or when the environmental temperature changes from the thermoneutral zone (below the low critical temperature) induces the response of peripheral thermoreceptors located at the dermal level. Transient potential receptors (TRP) TRPM8 and TRPA1 are activated at temperatures below 27 °C and 17 °C, respectively [34,35]. These TRP transduce the thermal stimulus and transmit through the primary sensory fibers, such as the A-beta, A-delta, and C fibers, which conduct the thermal sensation to higher brain structures, such as the hypothalamus, specifically in the POA, a structure that also receives thermal signals from the solitary tract [36].

The preceding allows the understanding that the successful compensation of hypothermia largely depends on the degree of neurodevelopment of the structures that coordinate the cited response.

Heat production is the generation of heat through intensified muscular activity or shivering, or the production of metabolic heat through the breakdown of BAT, which is generated mainly in the thorax or perirenal areas [12,14,22]. The regulation of shivering involves structures that connect the POA with the lateral parabrachial nucleus, the dorsomedial hypothalamus, the raphe pallidus, and motor neurons of the spinal cord [37]. Although shivering employs thermosensitive neurons in the POA and the spinal cord, these neurons also activate mechanisms such as breaking down BAT. Banet et al. [38] determined that the POA activation threshold is higher than for shivering in the case of BAT activation, whereas the opposite case occurs in neuronal cells of the spinal cord, in which the threshold for shivering is lower than for breaking down BAT. The above means that when a newborn requires activation of BAT to produce heat, the neurons of the POA will be the first to respond; in contrast, if the required mechanism is shivering, the neurons of the spinal cord will play a primary role. 

**Figure 1:**
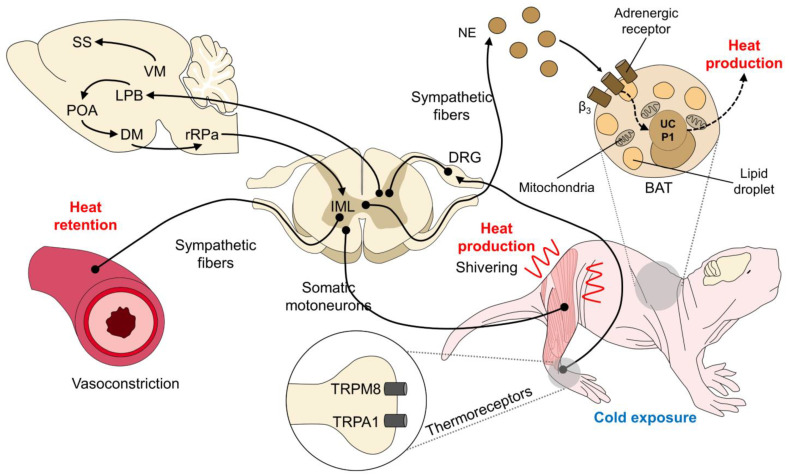
Neonate compensatory mechanisms in response to hypothermia. In newborns (illustrated by a naked mole (*Heterocephalus glaber*) in this figure), when the peripheral receptors responsible for the thermal sensation of cold are activated (e.g., TRPM8 and TRPA1), a neuronal response is generated that involves spinal structures (DRG) and the brain. In the brain, the thermoregulatory center (POA) receives the signal from the LPB. The POA has connections to the DMH, which, in turn, is connected to the rRPA and the IML neurons. Once in the spinal cord, two responses are produced through sympathetic efferents. Firstly, the innervation of blood vessels generates vasoconstriction and heat retention; conversely, the sympathetic release of NE acts on the BAT adrenergic receptors to produce heat. An additional response is shivering, a heat-generating process that depends on the spinal cord’s somatic motoneurons and their terminals. These mechanisms promote the production or retention of heat to protect the body from the consequences of hypothermia. BAT: brown adipocyte tissue; DMH: dorsomedial hypothalamus; DRG: dorsal root ganglion; IML: intermediolateral nucleus; LPB: lateral parabrachial nucleus; NE: norepinephrine; POA: preoptic area of the hypothalamus; rRPa: rostral raphe pallidum; SS: somatosensory cortex; UCP1: uncoupling protein 1; VM: ventromedial hypothalamus.

Additionally, it is mentioned that the presence of insulator fur makes animals less susceptible to thermal losses than those with glabrous skin, such as human or pig newborns. In them, there is a greater heat production capacity per unit of body weight and a smaller surface area about its weight [1]. However, BAT thermogenesis causes considerable energy consumption, and the newborn resorts to the use of glucose and oxygen reserves to produce heat, with the consequent risk of hypoxia, adynamia, hypoglycemia, and even death, emphasizing the importance of early recognition of hypothermia in the neonate [39] (Figure 1).

Another hypothalamic coordinated thermogenic response begins with reducing heat loss through peripheral vasoconstriction before catecholamine neurosecretion (adrenaline and noradrenaline). Vasoconstriction preserves the central temperature in organs with high metabolic requirements, such as the brain [1]. Furthermore, in the case of the newborn piglet, decreasing blood flow through vasoconstriction of the nearby skin capillaries helps decrease corporal heat loss during convection exchange [25,36].

The thermoregulatory capacity during the postnatal stage will primarily be dependent on the NS response to activating thermogenesis mechanisms and whether the species is altricial or precocial. In both, the compensation strategies depend on the NS maturation degree and the presence or absence of structures such as hair or fur or BAT activation, which will be discussed in detail in the following sections. 

## 3. Morphoanatomical Differences Associated with Thermoregulation in Precocial and Altricial Newborns

Neonates of precocial species are physically mature at birth, with fully functional ears and eyes, fur-bearing [40], and do not require constant parental care or nest rearing. The locomotion capacity of the precocial allows them to move and to immediately seek the dam’s udder and start suckling after birth [41,42]. Being animals that show a greater organic development, essential vital functions such as thermoregulation are facilitated [40] since the organogenesis of structures such as the lung, liver, and brain are carried out in utero [43].

**Figure 2:**
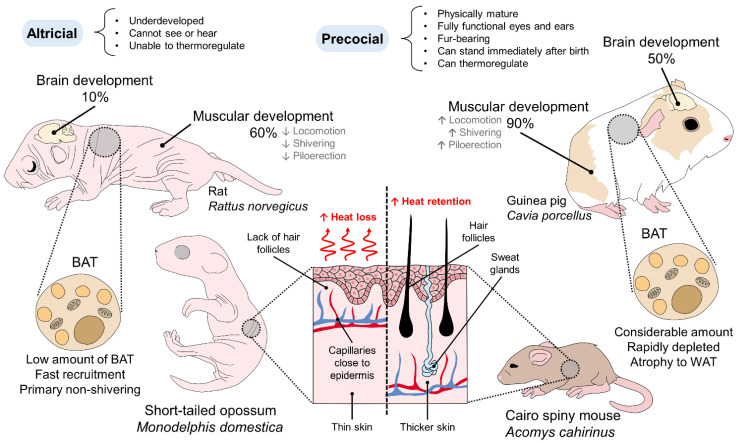
Morphoanatomical differences in altricial and precocial newborn rodents and their influence on thermoregulation. In altricial or underdeveloped *Monodelphis domestica* and *Rattus norvegicus*) and precocial or physically mature species (*Cavia porcellus*, *Acomys cahirinus*), morphological characteristics promote or hinder thermoregulation. The lack of fur, low amounts of BAT, uncoordinated locomotion, and thin skin with capillaries close to the epidermis contribute to heat loss and susceptibility to hypothermia in altricial species, contrarily to precocial species. BAT: brown adipose tissue; WAT: white adipose tissue.

In contrast, the offspring of altricial species are small-sized, compared to precocial species, naked with little or no fur, with uncoordinated locomotion (Table 1). If they are removed from the nest or the parents, or the parents are unable to feed them, their thermoregulation is affected because they cannot produce sufficient endothermy heat, resulting in hypothermic mortality [44]. However, enhanced postnatal plasticity is attributed to altricial animals. For example, altricial species are born with incomplete maturation of the nervous system and organs (e.g., the lungs) and a low metabolic rate, but these characteristics are completed faster than precocial animals during the first days of birth [45]. Nevertheless, because this process takes days, they lack thermoregulatory capacity immediately after birth, and they are susceptible to hypothermia [40]. Lambs have shown that their thermoregulatory ability is present within the first 60 minutes after birth, unlike other precocial species such as cattle calves or the altricial, such as puppies [5]. This difference within precocial species arises from the physiological and morphological resemblance to altricial species, when animals have reduced thermoregulatory capacity at birth, such as piglets without an adequate amount of BAT, or newborn mice and rat pups, in which the maturation of BAT coincides with the full functionality of the HPA, around seven days after birth (Table 1) [46,47]. A similar case is seen in neonatal European rabbits (*Oryctolagus cuniculus*) in which the expression of UCP1 depends on the environmental challenges to thermoregulate, not only recurring to metabolic pathways through BAT activation but behavioral changes such as huddling [48]. Figure 2 schematizes a comparison between altricial and precocial rodents and the morphological differences that influence thermoregulation. 

Neurologically, differences in brain growth and development between precocial and altricial species are observed. The central nervous system maturation in altricial animals depends mainly on the postnatal period because the hypothalamic structures are the main thermoregulatory organs. During their fetal state, the brain reaches only 10% of its total growth, in contrast to precocial animals in which it reaches up to 50% of its adult size [41]. In rats (*Rattus norvegicus*), maturation of the prefrontal cortex, with complete synaptogenesis and myelination, occurs at approximately 90 days post-birth [49]. As a result, delayed neural processing in altricial species delays the development of sensory and behavioral skills that influence their ability to thermoregulate [41]. 

The degree of muscle development, one of the thermoregulatory characteristics that generate the shivering mechanism in newborns, is another factor that presents differences between the offspring of altricial and precocial species [64]. Grand [41] has recorded the force-to-weight ratio in the altricial and the precocial as 60% and 90%, respectively, meaning greater motor and thermoregulatory independence in the latter. In the altricial, the muscular resources for heat production, such as locomotion, piloerection, and shivering are immature and do not participate until after three weeks of birth [65]. 

Similarly, skin thickness and the presence or absence of fur are relevant for thermoregulation, and they present differences between altricial and precocial animals. In altricial species such as gray short-tailed opossum (*Monodelphis domestica*), the skin in which the receptors in charge of perceiving thermal stimuli are located [66,67,68] has a thinness of 63.2 ± 2.2 μm [40], with an average range among marsupials of 36 to 186 μm [69]. In these animals, many superficial capillaries are located near the epidermis, between 38.5 ± 1.4 μm for *M. domestica* [40] and 30 to 35 μm in the case of the quokka wallaby (*Setonix brachyurus*), an extreme case of altricial species, facilitating heat loss [70]. Additionally, there is a complete absence of sebaceous and sweat glands and hair follicles [68], which, coupled with the small body size, makes them susceptible to hypothermia, relying entirely on their mother for thermoregulation. Additionally, the lack of insulator fur increases the area-to-mass ratio and susceptibility to heat loss [71]. This ratio is known as an association where, for example, small animals have a relatively larger surface area from where they can lose or dissipate heat with respect to their volume, compared to larger animals [72]. Unlike precocial species such as guinea pigs *(Cavia porcellus)* or the African spiny mouse (*Acomys cahirinus*) [43] in which the young are born with fur, achieving a maximum heat production rate of 70 ml/kg/min at ambient temperatures of 30 °C [73]. In lambs, heat production depends on shivering and non-shivering thermogenesis. The ability of lambs to cope with cooling environments has shown differences between individuals and may be related to genetic and phenotypic factors such as properties of the coat (wool), body weight, and skin. Body weight would be particularly important because of the effects and relationship between surface area and volume ratios [22]. 

Lastly, the primary mechanism of heat production in neonates is BAT activation; however, the perinatal development of BAT differs among species. Precocial animals such as guinea pigs, lambs, and cattle calves, are born with a well-developed BAT that atrophies rapidly, and it is replaced by white adipose tissue (WAT) within a short time during the postpartum period [74,75]. In contrast, altricial species are born with little or no BAT, whose deposition increases when exposed to low temperatures during the first weeks of birth [75], and they are the primary source of metabolic heat [66]. In mice (*Mus musculus*), this happens at approximately two weeks of birth [76]. Analysis from days 0 to 120 after birth has reported that the interscapular BAT’s weight increases from approximately 10 mg to almost 60 mg, respectively, to be replaced later by WAT [77]. This replacement or atrophy of BAT depends on the species. In rabbits (*O. cuniculus*), ovine (*Ovis aries*), bovines (*Bos taurus*, *Bos indicus*), and goats (*Capra hircus*), BAT disappears within a month, from 2 to 3 days in rabbits and ovine and from 2 to 6 days after birth in bovines [75]. A particular case among precocial species is in pigs (*Sus scrofa*). Despite having independent locomotion within a few minutes of birth [78], piglets are highly susceptible to hypothermia due to their large surface/body mass ratio [56], their little subcutaneous fat, and the fact that they are born wet and hairless [79]. In particular, the wet surface of newborns due to amniotic or placental fluids can promote hypothermia, as described in piglets by Malmkvist et al. [80], influencing or enhancing the cooling effect on the skin and heat loss by evaporation immediately after birth [81].

To sum up, the morphological characteristics of the animals at birth will dictate their thermoregulatory capacity, either facilitating or altering it. For this reason, it is necessary to understand the specific precocial and altricial mechanisms used to preserve body temperature before we introduce any intervention to prevent neonatal hypothermia. 

## 4. Thermoregulatory Mechanisms in Altricial and Precocial Species

### 4.1. Brown Adipose Tissue Activation (BAT)

Among the three types of adipose cells found in mammals (brown, white, and beige), BAT represents the least abundant but the one with the greatest thermogenic capacity, especially at birth [82]. Nonetheless, its quantity depends on the fetal development during gestation, the species (altricial or precocial), and the deposited anatomical site [83]. 

Unlike WAT, BAT has more mitochondria, high cytochrome Cc content, a vast vascular network [22,84], and BAT activation is mediated by uncoupling protein 1 (UCP1) [8]. UCP1s respond to the secretion of norepinephrine (NE) released by the sympathetic nerves and its action on β3 adrenoreceptors, whose effect is reversible through vagal stimulation (parasympathetic) [11,85]. Among other actions, NE promotes the proliferation of preadipocytes, the differentiation of mature adipocytes, regulates the expression of genes that encode UCP1, and increases mitochondrial mass. Altogether, NE actions contribute to heat generation through ATP synthesis by UCP1 [86] and lipolysis [87]. In the case of altricial species such as laboratory rodents, the relative percentage of BAT and white adipocytes can vary, depending on environmental and nutritional conditions, sex, and age. However, its remarkable plasticity allows retroperitoneal WAT to transform to BAT when exposed to cold [88]. 

An example of this was reported in 27 newborn deer mice (*Peromyscus maniculatus*) kept at a temperatures of 5 °C. BAT utilization increased by 42% (measured in terms of oxygen consumption), suggesting it is the only mechanism responsible for maintaining thermal stability during the first days of life [89]. Likewise, prenatal exposure to temperatures of 15 ± 4.2 °C on a female Darwin´s leaf-eared mouse (*Phyllotis darwini*) was shown to improve the thermoregulatory capacity of neonates, achieving higher body temperatures (32.3 ± 2.41 °C) compared to animals acclimated to an ambient temperature of 30 °C, which can be attributed to higher amounts of BAT and the increased expression of UCP1 in adipocytes [45]. Nevertheless, the characteristics and the properties of BAT may differ between lines of the same species. In a comparative study between B6 and A/J mice, it was found that cold stress only induced BAT activation in A/J mice due to genetic variability in the expression of UCP1 and adipogenesis. B6 mice are an inbred strain used to study obesity, a trait associated with BAT [90], while A/J mice are another strain with susceptibility to obesity and, together with B6 mice, have shown regional differences after adrenergic stimulation of UCP1 [91]. In B6 mice, a resistance of BAT induction has been reported by adrenergic stimulation, contrary to the A/J strain. In A/J mice, the UCP1 expression in the retroperitoneal fat at 60 days of age was higher than in B6 mice, with an induced activity of 71%, more active than interscapular BAT. In contrast, in B6 mice, the presence of BAT was lower than that found in A/J mice at one month of birth [77]. 

Additionally, the mother’s diet and body condition have also been associated with the functionality and pre- and post-natal development of BAT. A study with female C57/BL mice of 10 to 12 weeks of age observed that obese mothers fed a high-fat diet presented a deficient activity of BAT as a thermoregulator, where the activation and the expression of UCP1 and other proteins responsible for lipolysis had a lower oxygen consumption. In contrast, a deficient BAT activation was not observed in mice from dams with balanced diets [92].

On the other hand, species that are born with a low birth weight in relation to the average birth weight of the species or breed, like canine puppies, are more exposed to hypothermia because they have less adipose tissue. Moreover, when there is competition with littermates for access to a nipple/teat or a deficiency in colostrum intake at birth, there is a higher risk of hypoglycemia, which has important repercussions on neonatal survival [93,94]. 

Precocial animals, such as ruminants usually present a greater development of thermoregulatory mechanisms at birth, allowing them to maintain a constant body temperature, even in cold environments [30]. In these species, non-shivering thermogenesis is the most used mechanism in neonates. For example, in lambs (*O. aries*), approximately half of the cold-induced summit metabolic rate comes from non-shivering thermogenesis. The presence of metabolic-active BAT during the early postnatal period is essential [95]. However, adipose tissue distributed in the pre-scapular, inguinal, and prerenal regions represents only 2% of the total body weight [85,96].

The thermogenic activity has been measured in perirenal adipose tissue from newborn lambs (*O. aries*) for up to 33 days. In these animals, the impact of cold acclimatization of the pregnant dams can influence the thermogenic capacity of the offspring. In lambs from mothers exposed to cold climates, they had a 21% greater perirenal fat, increased metabolic activity (40%), and higher oxygen consumption in cold temperatures (16%). Additionally, the thermogenesis responses of these lambs were due solely to non-shivering thermogenesis, in contrast to lambs from dams not climatized to the cold [97]. The activation of BAT tissue responds to an increase in blood levels of cortisol, NE, and epinephrine. These catecholamines bind to beta-3-adrenergic receptors located in BAT, activating the UCP1 in the inner mitochondrial membrane. UCP1 and thermogenin increase the H+ ion flux at the mitochondrial level without ATP production [35]. Similarly, during birth, the plasma levels of hormones such as triiodothyronine (T3) and thyroxine (T4), triggered by the release of thyroid-stimulating hormone (TSH), increase metabolic consumption of adipose tissue to produce non-shivering thermogenesis [29]. In lambs, Schermer et al. [98] studied the thermoregulatory capacity of newborn lambs with fetal thyroidectomy. According to Litten et al. [99] and Silva [100], the thyroid hormone pathway for heat production is more developed in precocial species. Thyroid hormones are critical for the generation and the maintenance of body basal temperature (BBT), and even slight changes in hormone levels can affect BBT [100]. It has been observed that minor changes in thyroxine (T4) concentrations significantly impact body temperature [100,101]. BAT contains multiple enzymes called deiodinases, essential for converting T4 to active triiodothyronine (T3). In other words, BAT can generate T3, which is crucial for producing ATP and heat [102]. When exposed to cold stimuli, the enzyme 5-deiodinase type II is activated, converting T4 to T3. However, if T3 is not produced, UCP1 synthesis is blocked, leading to hypothermia [103].

Due to the influence of thyroid hormones in thermoregulation [99,100], thyroidectomized animals presented lower colon temperatures (up to 2.35 °C) than control animals. For example, Berthon et al. [104] found that in pigs, lower plasma levels of T4 are present in animals with a lower rectal temperature after birth. Likewise, the thyroidectomized animals had a lower oxygen consumption rate and a higher incidence of shivering thermogenesis, which coincides with a lower activity of the perirenal adipose tissue, lower levels of uncoupling protein, and a higher lipid content.

In most mammals, concurrently with non-shivering thermogenesis, colostrum intake in the first hours of life represents an energy resource that contributes to maintaining a stable temperature in neonates [2]. In particular, the nutrients present in colostrum provide water, bioactive compounds, growth factors, digestive enzymes, and immunoglobulins, and one of its main roles during the first days after birth is the supplement of energy in the form of kcal/L. Although it is said that the nutritional properties of colostrum and milk are similar during the first days of life, the energy value of colostrum can be 20–30% higher than the values registered after three days or two weeks [105]. Additionally, colostrum intake and glucose absorption prevent hypoglycemia due to the low fat and glycogen storage in newborns [106], maintaining normal glucose levels that can support thermogenesis [107]. For example, in calves, colostrum provides large amounts of glucose and amino acids, equivalent to 6.7 MJ/g, that can be used to produce heat [29,108]. Piglets (*S. scrofa*) are a species born with low amounts of BAT; their main ways of heat production rely on shivering and colostrum intake shortly after birth [23]. Furthermore, several molecular (e.g., the presence of uncoupling proteins 1, 2, or 3, the responsible for non-shivering thermogenesis), ultrastructural (e.g., number of mitochondria in *longissimus thoracis* and *rhomboideus* muscle per unit tissue area), biochemical (e.g., fat oxidation, mitochondrial processes), physiological, and metabolic adaptations in the maturation of the energy production of the musculoskeletal system in piglets are an adaptive thermoregulatory mechanism [23]. Additionally, newborn piglets use their body fat and glycogen stores to survive in the first 12 to 24 h after birth [109].

On the other hand, the fetal development of BAT, the mother’s diet during gestation, and the influence of hormones such as melatonin have been shown to influence the thermoregulation capacity of the newborn. The case of 5 to 6-day-old lambs born from dams with low melatonin profiles and exposed to 4 °C showed a reduction in BAT temperature of approximately 39.8 °C compared to the control group of 40 °C and elevated NE concentrations greater than 1000 pg/ml, as a result of thermal stress [110]. 

Thermogenesis by BAT activation is essential in the neonate of most species, and it is also essential in hibernating animals such as American black bears (*Ursus americanus*) [111,112], which represents the first resource during the postnatal period. However, for species with limited energy reserves at birth, such as newborn piglets with low amounts of BAT or rodent pups with non-fully developed interscapular BAT, colostrum intake and other mechanisms to preserve heat are critical to prevent hypothermia.

### 4.2. Shivering

Shivering is the universal thermogenic mechanism through the repetitive and rapid contraction of the skeletal muscle when the body is exposed to cold environments or when there is hypothermia. The muscle fibers are from resistant aerobic muscles that can produce repeated contractions [113]. This process utilizes the oxidation of carbohydrates, lipids, and proteins obtained from muscle reserves and the circulating blood [114].

Despite being a mechanism for heat production, shivering includes consequences such as an increase in oxygen consumption of up to 20-fold, increasing the aerobic capacity of muscle fibers, and leading to fatty acid oxidation [113]. Other consequences include an increased intracranial pressure and metabolic demand, causing poor ventilation synchronization [115]. This hypoxic effect has been reported in newborns. For example, in deer mice (*P. maniculatus*), high-altitude habitats (4350 m.a.s.l.) reduce the capacity to generate heat by shivering. The capacity is reduced by 30%, and only when deer mice pups reach 27 days-old do they develop an aerobic muscle phenotype, predisposing them to hypothermia and mortality. However, this characteristic is also considered a physiological adaptation to reduce energy expenditure by thermoregulation [116].

One of the main differences in thermogenic capacity through shivering between altricial and precocial species is caused by species-specific morphological features. For example, the intensity of shivering depends on the percentage of body fat, the surface-to-volume ratio, and the ATP necessary to maintain contractions [114]. In the case of dogs, the mechanism of shivering thermogenesis is poor or absent, having a greater risk of hypothermia. Additionally, dog puppies (*Canis lupus familiaris*) have only 1.3% body fat [117]; therefore, they rely on milk intake and constant maternal care to properly thermoregulate [118,119]. A similar case is seen in cubs of polar bears (*Ursus maritimus*), where they are born in temperatures as low as −25 °C. Over time they increase the ability to shiver, improve their insulation traits and, in some cases, develop BAT [120], or resort to other methods such as vasomotor control to maintain an adequate body temperature.

As stated before, thermogenesis through rapid and oscillating muscle contractions or twitching of skeletal muscle is an involuntary mechanism that produces energy released in heat [35]. Although for most precocial species it is the most efficient method for heat production and thermal equilibrium when exposed to cold environments [50], it cannot be used as a primary method due to the immaturity of the muscle tissue observed in some species, such as ruminants) [30].

In this sense, shivering heat production in piglets has been associated with a decrease in muscle glycogen up to 47%, as well as a decrease in total lipid content, a decrease in lactate in blood, and better muscle cytochrome oxidase activity (by 20% more), indicating the increase in muscle potential with exposure to cold [121].

According to Alexander and Williams [122], in one-day-old Merino lambs and one-month-old lambs, the mechanisms of thermogenesis by shivering, in comparison with the mechanisms that use BAT, are considered the basis of its thermoregulation in the first days of life. Similarly, shivering is considered a complementary mechanism activated in the first four days of birth because newborns suffer rapid thermoregulatory alterations when the adipose tissue is insufficient to maintain thermal comfort [97].

On the other hand, in piglets exposed to low temperatures (25 °C), thermogenesis by shivering increased its activity. However, their temperatures remained slightly lower than newborns exposed to thermoneutral temperatures (34 °C) [121]. In a recent study, Schmitt et al. [123] evaluated the piglets’ thermoregulation efficiency from two divergent lines for the residual feed intake (high feed efficiency and less feed efficiency). Rectal temperature, infrared thermography of ear base and tip and back were recorded. Vigor, evaluated by respiration, mobility, vocalization, and morphology, was also registered by weight, length, width, and circumference. The authors observed that both vigor and morphology did not vary between piglet lines, but it was possible to observe a greater weight gain in the efficient lines (7.1 ± 1.3 g) compared to the less efficient (3.6 ± 1.3 g). Likewise, animals with a higher efficiency had a lower temperature in the ear region (24.7 ± 0.37 °C vs. 26.3 ± 0.36 °C). All of the above allows us to establish that thermogenesis through shivering is a mechanism that depends on the type of muscle fiber of the newborn. In this process, efficient feeding is essential since neonates use their feed intake as a source of energy to maintain vital function and, consequently, survival. However, if the source of hypothermia is not addressed, continued shivering can have adverse consequences for the neonate.

### 4.3. Vasomotor Control

Another sympathetic-dependent response to hypothermia is vasomotor shifts in the peripheral circulation [124]. When exposed to a cold stimulus, the cold-sensitive neurons located in the POA and the activation of the HPA axis induce the secretion of catecholamines (epinephrine and NE) and other neurotransmitters such as neuropeptide and ATP [35]. Consequently, they activate receptors in the blood vessels to produce vasoconstriction [125,126] to divert blood flow from the limbs or peripheral structures to vital organs [127,128,129]. Solomon et al. [130] demonstrated this in four Long Evans rats (*Rattus norvegicus*) subjected to motility frustration (e.g., not being able to use an activity wheel). These animals showed restlessness, and the stress caused low paw temperatures due to sustained vasoconstriction.

In precocial species, some differences in thermoregulation between breeds have been reported. In pigs (*S. scrofa*), 2 to 4-hour-old Meishan piglets (*Sus domesticus*) have greater development at birth than piglets of the European breed. For example, in Meshian piglets, cardiovascular responses to cold (vasoconstriction) were observed on birth/1 day/2 days after birth. In contrast, in Pietrain, Landrace, and Large White crossbred piglets, the vasomotor response capacity was not observed until five days after birth [56]. However, these differences cannot only be associated with the vasomotor response. Renaudeau et al. [131] have studied the thermoregulatory differences between European (Large White) and Caribbean (Creole) pigs regarding breed, season, and skin histology. The authors found that the dermis of Creole pigs was thicker than Large White (3.60 vs. 3.13 mm) and they had a higher density of sweat glands (32.0 and 25.4 glands per mm2, respectively). Although these traits can be associated with enhanced adaptability of Creole pigs as a heat-tolerant breed, they may also influence the thermoregulatory efficiency in piglets during the first days of life or the growing stage, but this research needs further studies [132]. As a possible assessment of this vasoconstriction during hypothermia, infrared thermography (IRT) has been implemented, which reflects the peripheral blood flow through the emitted radiation [133].

Kammersgaard et al. [134] evaluated the thermal response in 91 newborn piglets under three different environmental temperatures (15 °C, 20 °C and 25 °C) through IRT and rectal temperature from birth to 48 h after parturition. They observed a positive correlation between the ear and rectal body temperature. For example, when the piglet’s IRT indicated a temperature of 30 °C, the rectal temperature was 32 °C or less, with an IRT confidence of 91.3%. Similarly, McCoard et al. [135] evaluated the thermal response by IRT and rectal temperature after birth in 10 newborn lambs. Continuous thermograms were recorded during the evaluation of a 30-min sequential baseline (11–18 °C), 30-min cold exposure (0° C), and 30-min recovery (11–18 °C) time evaluation. They observed that the rectal temperature decreased between 0.4–1 °C from the baseline to the end of the recovery period, while there were no changes in IRT during the baseline event. Five minutes after cold exposure, a rapid decrease of 5 °C was observed. These authors attribute that the observed linear thermal response is due to the change in surface blood flow in response to cold to preserve heat. This result makes it possible to suggest IRT as a valuable tool because of its non-invasive nature and the correlation between the decrease in peripheral blood flow caused by hypothermia.

### 4.4. Behavior and Postural Changes

Besides the metabolic and physiological mechanisms of thermoregulation, animals perform certain behaviors and postural changes to minimize heat loss [136]. Some examples are warmth seeking, nesting, burrowing, huddling, basking, and calling for the mother [40,137]. 

**Figure 3:**
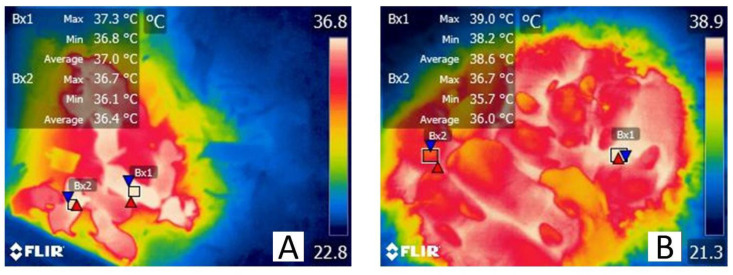
Huddling behavior in newborn Wistar rats (*R. norvegicus*) and New Zealand rabbit pups (*O. cuniculus*). (**A**) Thermoregulatory effect of huddling and the influence of the position within the nest in 1-day-old rat pups. The surface temperature of one of the rats in a central position, measured in the dorsal interscapular region (Bx1), shows a maximum, a minimum, and an average temperature of 37.3, 36.8, and 37.0 °C, respectively. In contrast, the temperatures of one rat pup in the nest periphery (Bx2) registered a maximum value of 36.7 °C, a minimum of 36.1 °C, and an average of 36.4 °C. (**B**) 1-day-old New Zealand rabbit pups huddling with conspecifics (Bx1) in a central position are observed as a white tone in the thermogram. A reddish-yellow tone is observed in newborn rabbits positioned in the group periphery (Bx2). Between central and peripheral positioned rabbit neonates, a difference of up to 2.3 °C in the minimum temperature (39 vs. 36.7 °C) and an average difference of up to 0.6 °C were recorded. These values measured through infrared thermography provide information on the importance of neonate rodents and leporids’ behaviors to preserve heat and to maintain a stable body temperature. The authors took the thermographic images with a FLIR E80 camera with an 18 mm FOL lens at a resolution of 320 × 240 pixels and the ability to accurately measure temperatures from −20 °C to 550 °C/−4°F to 1022°F (emissivity = 0.95, distance = 30 cm).

Most of the thermoregulatory behavioral changes observed in animals are innate activities. However, there is evidence that learning is another adaptative mechanism to adverse environmental conditions, as observed in rats who have shown their capability to learn and light lamps to generate heat [138]. It is believed that these behavior and postural changes involve the activation of the POA; however, it has not yet been established [11]. In pigs (*S. scrofa*), it has been observed that adopting postures such as huddling with littermates [139] or assuming a sternal position reduces the contact surface with the ground and prevents heat loss at birth [25]. In European rabbit kits (*O. cuniculus*), the most frequent behaviors are huddling, rooting, climbing, and maintaining close contact with the rest of the littermate. These behaviors seek to retain a better position within the nest, ensuring a source of heat and food [62]. In this same sense, García-Torres et al. [140] have studied the relationship between BAT, triglyceride concentrations, and huddling of the chinchilla-strain rabbits (*O. cuniculus*, *F. domestica*). In this study, the authors determined that BAT is the main activation mechanism of thermogenesis in newborn rabbits, and that posture changes are vital in preserving their body temperature. They reported that kits positioned on the group’s periphery had lower BAT reserves and low triglyceride concentrations (101.7 ± 24.8 mg/dL), suggesting that these animals were exposed to a greater thermal and metabolic challenge than the rabbit neonates found in central positions. IRT is a tool not limited to evaluating vasomotor thermoregulation mechanisms, and there are reports that this tool can record BAT or muscle activity in laboratory animals in different settings [141]. Figure 3 shows the author’s preliminary findings in concordance with those reported by García-Torres et al. [140] on the effect of huddling and animal position inside the nest. In this figure, Wistar rat pups and New Zealand rabbit (*O. cuniculus*) pups are shown to represent altricial species and the effect that central or peripheral position has on the superficial temperature of newborn rodents and lagomorphs. As stated previously, postural changes such as adopting a central position are a thermoregulatory mechanism that prevent heat loss and facilitate heat conduction by the presence of littermates and even the dam.

In precocial species such as lambs, behavior at birth greatly contributes to their thermoregulation. One of its first reflexes is standing from the floor and seeking the udder to suckle and to consume colostrum. Getting up off the ground reduces heat loss, while the vitality and they speed with which the newborn finds the nipple promote early colostrum intake necessary for energy production [2]. Unlike lambs, kids (newborn goats) are considered more sensitive to hypothermia during the first hours of life. Giannetto et al. [142] have reported in Maltese kids that the circadian system is the predominant mechanism for maintaining homeostasis after birth due to the development of this system and the genetic and phenotypic differences with lambs. In newborn piglets, vitality, and suckle capacity influence their survival rate after birth and determine their thermoregulation efficiency [143]. Together with animals’ weight and size, vitality influences thermoregulation [144]. Moreover, it has been reported in newborn moose (*Alces alces*) that, in addition to the amount of BAT present at birth, newborns require feeding an average of 8 times a day in 130 second sessions to obtain nutrients and to thermoregulate [145]. Similarly, blue foxes (*Alopex lagopus*) in artic environments can reduce heat loss through postural and behavioral changes, increasing metabolic heat production to prevent heat transfer from the core to the surface [146].

### 4.5. Diving Air-Breathing Marine Vertebrates

Air-breathing marine vertebrates need to maintain thermal homeostasis in an oxygen-limited aquatic environment. That is why these animals, thanks to their phylogeny and thermoregulatory adaptations, have managed to survive in environments with changing temperatures using morphological, physiological, and behavioral traits [147,148,149]. Sirenians, for example, are the only herbivorous marine mammals with relevant thermoregulatory implications. They have a very slow metabolism, limiting their capacity for thermogenesis and making them sensitive to cold [150]. Marine mustelids and ursides are also exposed to extreme climatological challenges. Small marine mammals such as otters (*Enhydra lutris*) inhabit places with cold temperature climates to subarctic waters, while polar bears (*Ursus maritimus*) live in the arctic [151]. Another example is penguins (*Aptenodytes patagonicus*), which with their adaptations can lower their abdominal and subcutaneous temperature to −25 °C and then return to their normal temperature through subsequent rewarming [152]. We can observe that all these species have something in common: their evolutionary adaptations that have allowed them to survive in extreme temperatures.

## 5. Opportunity Areas and Application of IRT as an Evaluation Tool to Help the Intervention of Animals Cope with Neonatal Hypothermia

**Figure 4:**
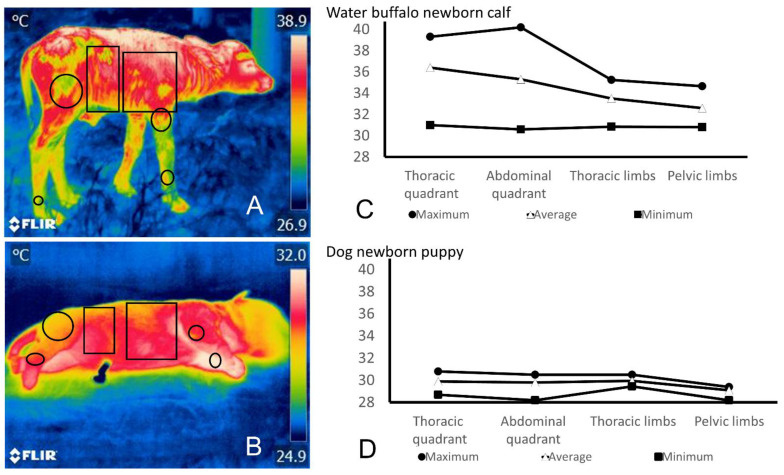
Thermographic evaluation in altricial and precocial neonates. (**A**) Dry newborn water buffalo calf (*Bubalus bubalis*) thermogram. The maximum temperature (shown as a white color in the thermogram) is observed in the facial and thoracic region, while the coolest temperatures (color green and yellow) are observed in the forelimbs and hindlimbs. (**B**) Dry newborn puppy (*Canis lupus familiaris*) thermogram. The maximum temperature can be seen in the cranial region of the thoracic and pelvic limbs (white color), while the back and the facial region (yellow color) and the nose (green color) of the newborn represent the coolest regions. (**C**) The graph exhibits the maximum, minimum, and average temperatures of the water buffalo newborn calf’s thoracic, abdominal, forelimb, and hindlimb areas. (**D**) The graph displays the newborn puppy’s maximum, minimum, and average temperature in the thoracic, abdominal, forelimbs, and hindlimb areas. A significant difference between the temperatures of both species can be recognized, where the water buffalo newborn calf has the highest values regardless of the evaluated region. Both thermograms were recorded at minute 60 post-birth to compare the thermoregulatory ability of a precocial animal (water buffalo neborn calf) and an altricial animal (puppy dog). The authors took the thermographic images with a FLIR E80 camera with an 18 mm FOL lens at a resolution of 320 × 240 pixels and the ability to accurately measure temperatures from −20 °C to 550 °C/−4 °F to 1022 °F (emissivity = 0.95, distance = 30 cm).

IRT has shown that it allows evaluating the thermal state of animals when exposed to extreme environmental conditions, but also in the newborn it allows monitoring each of the thermoregulatory mechanisms during hypothermia [25,134]. Its non-invasive and real-time monitoring properties make it an alternative for evaluating the body’s response to hypothermia in different species [153,154,155]. For example, Figure 4 shows a comparison between thermograms taken 60 min after birth in a precocial (water buffalo newborn calf) and an altricial animal (newborn puppy dog). According to their characteristics and neurodevelopment, precocial species such as water buffalo can stand up almost immediately after birth [41]. This ability prevents heat loss by contact with a surface, resulting in higher superficial temperatures, as shown in the buffalo thermogram. In contrast, at the same time after birth, altricial animals such as newborn dogs are not able to incorporate or actively seek the teat until day 21 [156], and their limited thermoregulatory capacity is reflected in the lower superficial temperatures shown in the B thermogram.

IRT has been suggested as a tool that could evaluate the compensatory thermal response to hypothermia in different species such as dogs, pigs, and ruminants [25,119,135]. In this sense, IRT could help to identify hypothermia in the newborn early and assess the continuous heat loss during the postnatal period. In the same way, it has been observed that IRT helps to evaluate the organism’s adaptation towards hypothermia in litters of female desert hamsters (*Phodopus roborovskii*). At birth, their surface temperature was similar to that of the environment (21 °C) due to their low body mass. In contrast, after 15 and 16 days, their body mass increased to 5.5 ± 0.2 g and 5.8 ± 0.2 g, respectively, facilitating metabolism and heat production [44]. The same effects have been observed in species that resort to a state of hibernation but cannot compensate for their temperature in environments where the cold is extreme. As a result, IRT has been suggested as a way to identify a state of severe hypothermia [157]. Some authors report the usefulness of IRT in assessing the thermal state of neonates [134,158], and some results show a positive correlation between thermal response and rectal temperature [36,123]. However, the results are not as conclusive. For example, it has been reported that ocular temperature has no association with rectal values [159]. Soerensen and Pedersen [160] mentioned that although the temperature of the base of the ear, eyes, and udder has a high correlation with rectal temperature, factors such as age and biological status can alter its reading. Therefore, IRT may not be able to accurately assess body temperature with precision, but it is considered a valuable tool to assess changes in superficial temperature [134,159].

IRT has also been used in maternity pens to evaluate the influence of the pen characteristics (e.g., ventilation systems) on the thermal response of sows and piglets. Dela Ricci et al. [161] found that ventilation and roof sprinkles did not reduce the superficial temperature of sows during summer, meaning that the roof design and the material were not providing an adequate heat flow. Additionally, Labeur et al. [162] have determined that the body region used to assess IRT in ewes shorn during pregnancy influences the neonate’s results. In lambs, 4 hours after birth, the maximum and the average temperatures were located in the hip, and it was found that lambs born from shorn ewes maintained an adequate surface temperature when compared to the control animals, suggesting a better development of BAT in these animals.

## 6. Conclusions

One of the situations that can affect the survival of newborns is low temperatures or cold environments since their thermoregulatory system is limited in most cases. Neonatal animals from altricial species are usually more likely to suffer from hypothermia due to their reduced maturity/development at birth. On the contrary, precocial animals tend to adapt more quickly to thermal changes at birth, except for individuals born with a lower birth weight or difficulties during parturition that cannot have adequate access to colostrum. The ability of newborn mammals to maintain their core temperature within normal parameters is fundamental for their adaptation to the extrauterine environment and survival. That is why understanding better the specific mechanisms used by each species will allow targeted interventions to develop. Moreover, implementing thermoregulation monitoring tools, such as IRT, would help to refine and to assess the success of targeted interventions to prevent hypothermia and the consequences it can generate in neonatal mammals.

## Figures and Tables

**Table 1:** Main mechanism of thermoregulation and its maturation time in different species. The difference between altricial and precocial species depends on the degree of neurodevelopment which can produce variations in time to achieve optimal thermoregulation between them. Precocial species reach this capacity in the first hours after birth, while altricial species develop this ability between 20 and 45 days after birth.

	Species	Maturation Time	Main Mechanism of Thermoregulation	References
Precocial	*Bos taurus* calves	1 to 2 h after birth	Non-shivering thermogenesis Shivering thermogenesis Vasomotor control Postural changes	[18,50,51]
	Piglets	4 to 8 h after birth		[25,52,53,54,55,56]
	Lambs	1 to 5 h after birth	Shivering and non-shivering	[22]
Altricial	Rat pups	13 to 20 days after birth	Postural changes	[57,58,59]
	Rabbits	9 to 11 days after birth	Vasomotor control	[60,61,62]
	Kitten	45 days after birth	Non-shivering thermogenesis	[63]

## Data Availability

Not applicable.
